# Observations on the Camelidæ

**Published:** 1880-10

**Authors:** William A. Conklin

**Affiliations:** Director Central Park Menagerie


### ART. XIX.—SOME OBSERVATIONS ON THE CAMELIDÆ.

BY WILLIAM A. CONKLIN,

DIRECTOR CENTRAL PARK MENAGERIE.

AS most readers are already familiar with the natural history of the camel family, I shall confine myself to a few general remarks on their habits and peculiarities.

Among the animals confined in menageries there are few more healthy or requiring less care than the camelidæ. This, doubtless, may be attributed in a measure to the fact that they have not been obliged to undergo any change of habits in their life, as this family, with the exception of the guanaco and vicuna, have always lived in a state of domesticity. During the rutting season the male camel is intractable, and not unfrequently dangerous to his keeper. At this time he ejects from his mouth a loose membrane like a bladder, filled with air, with which he makes a loud and guggling noise. During this season, also, the secretion contained in the glandular elevations on top of his head flows more freely. This secretion is of a dark oily appearance, is very offensive, and makes an indelible stain. In the East, only certain of the males are kept for stud purposes, the others being castrated as a measure of protection. The mode of copulation of the camelidæ differs from that of other ruminants in this, that the male runs the female down and forces her into a kneeling position (her natural one of repose) before coition takes place. The season in the female is of three days' duration, recurring every four weeks, and the male will serve her eight or ten times a day if allowed, while the heat lasts. Period of gestation is twelve months, one calf only being produced at a birth, while miscarriages are of very frequent occurrence. In some sections of the East, after the calf is born, it is immediately wrapped in cloths or blankets as a protection against cold; this measure of precaution is not necessary, but is simply a fanciful idea of the people in that country. The calf seldom suckles before it is twenty-four hours old, as the legs are yet too weak to support the body in an erect position. When born it stands three feet high, has the seven callosities distinctly marked, and the neck so short that it will not admit of the head reaching below the knee. It suckles for ten or twelve months, reaches its full size in about five years, and lives, it is said, forty to fifty years, there being on record a case of one in the Schonbrunn menagerie living twenty-six years. The camel's milk is of a gummy nature, and is said to be very nutritive, but we have the authority of Layard for saying that it does not make butter. The cross between the bachian camel and dromedary has but one hump, and is a mule. There is no curve of the vertebral column in forming the hump; it consists of a mass of flesh, which serves the animal as nourishment when deprived of food for any length of time. After the fat has been absorbed for this purpose the hump may fill up again, but never regains its erect position. The camel is almost as omniverous as the goat, showing a preference, however, for the thistles and prickly grasses. It consumes about as much food as a horse does, and drinks during the summer on an average six gallons of water per day, and during the winter months, about half that quantity.

The Camelidæ are the only family of the ruminants that possess upper incisor teeth, of which there are two, and it frequently happens while confined in menageries that the lower incisor teeth become so much elongated that pieces have to be broken or filed off. The South American species interbreed with one another, and never shed their wool, while those from the East shed their hair regularly every year about the month of August. Another peculiarity of this species is that when angry they will strike with their forefeet and eject a quantity of half-digested cud upon any one on whom they want to vent their spleen.

The greater number of camels now found in this country are descendants of those imported by the United States government in 1856 with a view to be used for carrying purposes previously to the building of the Pacific Railroad. A number were sent to Virginia in 1701, but the efforts to introduce them failed, as the animals died for want of proper care. They are considered the oldest mammals now living, as is proved by the fossil remains found in the miocene of the Sevalk hills. A number of llamas were imported to this city in 1857 for the purpose of utilizing their wool, hides and flesh, but the attempt to introduce this new industry had to be abandoned, as the animals were sold at auction at a loss to the importer.

The most serious disease to which the camels in the East are subject is the mange, caused chiefly by disregard of cleanliness. If this distemper is not quickly arrested it often proves fatal. When the first symptoms appear, the animal is immediately separated from the rest of the herd to prevent contagion, and tar is plentifully applied to the parts affected, the hair being first cut off. Several applications are necessary before the disease is cured. Another complaint of importance is inflammation of the lungs—pneumonia, which generally carries off the animal in a few days; purges of rancid butter and olive oil is the usual treatment for this malady, although we think this treatment might be improved upon.

As to diseases of camels in the new world, I am happy to say my experience is very limited, having had to deal with only two serious cases. One of these was an aged male camel which died from uræmia, brought on by a large calculus in the kidney. In this case there was retention of urine. No relief could be given to the camel by the use of the catheter, owing to the exceedingly small calibre of the urethra, the urine having to be drawn off by puncturing the bladder through the rectum.

The other case was a young male, a year old, who suffered from diarrhœa. Simple remedies were applied without effect, and the complaint not yielding to this treatment, laudanum injections and bismuth were tried and found ineffectual. These animals are subject during the spring of the year to boils which generally come about the head.

